# Successful Use of Salvage Bacteriophage Therapy for a Recalcitrant MRSA Knee and Hip Prosthetic Joint Infection

**DOI:** 10.3390/ph15020177

**Published:** 2022-01-31

**Authors:** Jonathan Schoeffel, Elizabeth Wenqian Wang, Dustin Gill, Joseph Frackler, Bri’Anna Horne, Theodore Manson, James B. Doub

**Affiliations:** 1 Department of Orthopedic Surgery, University of Maryland St. Joseph Medical Center, Towson, MD 21204, USA; jschoeffel@towsonortho.com (J.S.); tmanson@towsonortho.com (T.M.); 2 Division of Infectious Diseases, University of Maryland St. Joseph Medical Center, Towson, MD 21204, USA; Elizabeth.Wang@umm.edu; 3 Department of Pharmacy, University of Maryland St. Joseph Medical Center, Towson, MD 21204, USA; DustinGill@umm.edu; 4 Adaptive Phage Therapeutics, Gaithersburg, MD 20878, USA; jfackler@aphage.com (J.F.); bhorne@aphage.com (B.H.); 5 Division of Clinical Care and Research, Institute of Human Virology, University of Maryland School of Medicine, Baltimore, MD 21201, USA

**Keywords:** bacteriophage therapy, periprosthetic joint infections, MRSA, biofilm

## Abstract

Prosthetic joint infections are a serious complication of joint replacement surgery due to the significant morbidity and financial burden that is associated with conventional treatments. When patients fail the gold standard two-stage revision surgery, very limited, well-defined standardized approaches are available. Herein, we discuss the case of a sixty-four-year-old woman who had a recalcitrant MRSA prosthetic joint infection of her knee and hip that failed repeated conventional surgical and medical treatments. Only after receiving intraoperative and intravenous bacteriophage therapy was the patient able to achieve cure of her prosthetic joint infections, as demonstrated by the lack of clinical recurrence and sterility of intraoperative cultures while off antibiotics. This case reinforces that bacteriophage therapy holds promise in the treatment of prosthetic joint infections and more specifically in complicated cases who have failed conventional surgical and medical interventions.

## 1. Introduction

Prosthetic joint infections (PJIs) are one of the most dreaded complications of joint replacement surgery, given the increased morbidity and mortality correlated with treating these infections [1]. Bacterial biofilms, which form on prosthetic surfaces and devitalized bones, are a major reason these infections are challenging to treat [2,3]. Consequently, revision surgeries are needed to cure chronic PJIs, in which biofilm-laden prostheses are removed in combination with prolonged antibiotic therapies. When patients fail the gold standard two-stage revision surgery, limited standardized options remain to cure these recalcitrant PJIs. Therefore, novel therapeutics are desperately needed to help cure chronic PJIs and thereby reduce the morbidity, mortality and enormous costs associated with these infections.

One such therapeutic is bacteriophage therapy, given its ability to infect and lyse biofilm bacteria, as well as enzymatically degrade the biofilm extracellular polymeric substances [4,5]. As a result, bacteriophages have been proposed as possible adjuvants with debridement, antibiotics and implant retention surgery (DAIR) to cure PJIs without prosthesis removal [6]. Although treating PJIs without prosthesis removal would revolutionize PJI treatment, the use of bacteriophage therapy with complicated two-stage revision surgery also has a potential role. Herein, we discuss a case of a patient who had a methicillin-resistant *Staphylococcus aureus* (MRSA) PJI of her hip and knee that conventional antibiotics and numerous revision surgeries were not able to cure. Only after bacteriophage therapy was a sustained clinical and microbiological cure achieved.

## 2. Case

A sixty-four-year-old woman underwent primary right hip and knee arthroplasty in 2018 for progressive osteoarthritis. She was in good health until June 2020, when she was admitted to a local hospital with right foot cellulitis and a dorsal foot abscess. The abscess was debrided, and cultures grew MRSA, prompting treatment with intravenous (IV) vancomycin followed by oral linezolid to complete a 14-day course. Shortly after stopping antibiotics, she developed right knee swelling, erythema and pain and was admitted to our hospital. 

**Figure 1:**
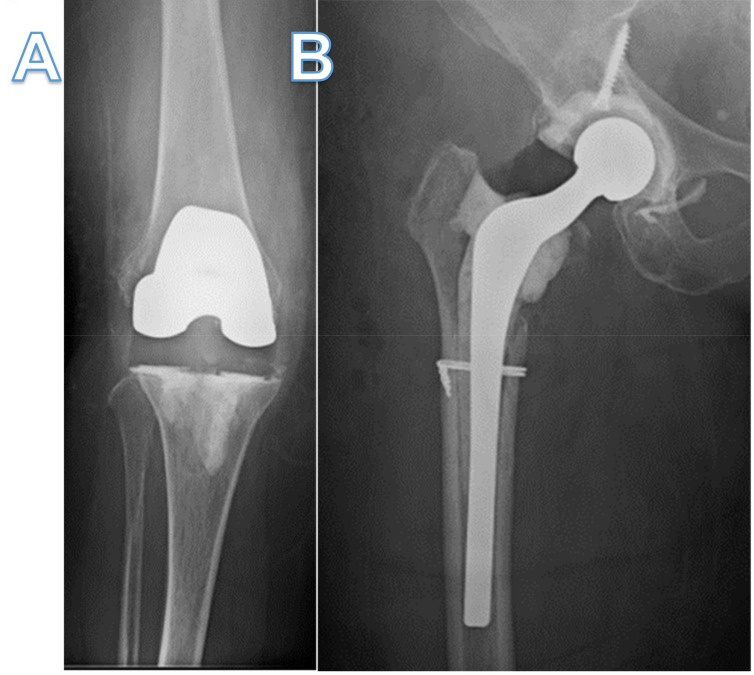

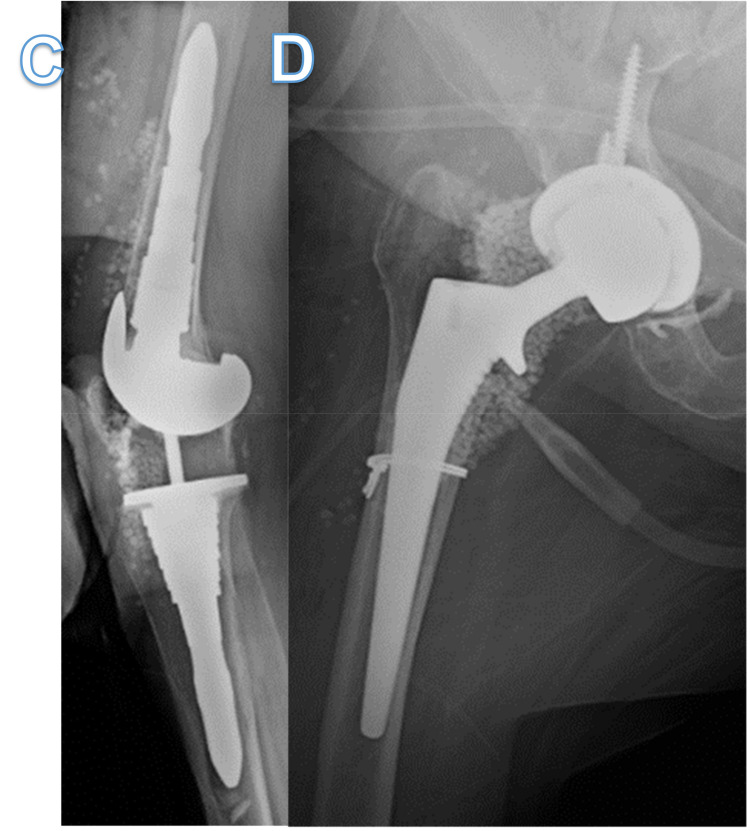
X-ray of knee and hip before and after bacteriophage therapy. (**A**,**B**) X-ray anterior-posterior view of right knee and hip showing temporary antibiotic-coated knee prosthetic and Prostalac hip spacer with residual cerclage wire and screw. (**C**,**D**) X-ray lateral view knee and anterior-posterior view hip showing total knee and hip arthroplasties implanted after bacteriophage therapy and subsequent proven sterility of the joints. Retained cerclage wire still present.

Arthrocentesis of her knee grew MRSA, prompting revision surgery with explant of prosthesis and insertion of a temporary implant with high dose vancomycin and tobramycin-loaded cement (Figure 1A). Despite being treated with vancomycin, she developed right hip pain in which a subsequent arthrocentesis grew MRSA. She then underwent DAIR of the right hip and was discharged on IV daptomycin 500 mg daily and oral rifampin 600 mg daily, but rifampin was later discontinued 7 days later due to intolerance.

Her right hip pain, swelling and erythema recurred while on daptomycin, prompting a two-stage revision with insertion of an antibiotic-loaded PROSTALAC (Depuy-Synthes, Warsaw, IN, USA) hip spacer (Figure 1B). She was then treated with daptomycin 500 mg daily for another six weeks, after which antibiotics were stopped. Two weeks after stopping antibiotics, she presented again with right knee pain, swelling, erythema and systemic signs of sepsis. An arthrocentesis culture grew MRSA. Due to hemodynamic instability, she first had irrigation and debridement of the knee, followed by an exchange of the antibiotic-impregnated temporary knee prosthetic to a new one four days later. Given the refractory nature of her infection, two Hickman catheters were inserted for intra-articular vancomycin dosing. She was then restarted on IV daptomycin as well as twice-daily intra-articular vancomycin. Transesophageal echocardiogram was negative for endocarditis, and computed tomography (CT) of the chest, abdomen and pelvis did not reveal any other foci of infection. 

Despite aggressive treatments, she continued to have a persistent MRSA infection of both joints. After extensive discussion with the patient, personalized bacteriophage therapy was pursued and, her clinical isolate was sent to Adaptive Phage Therapeutics (Gaithersburg, MD, USA), where a strictly lytic bacteriophage (SaWIQ0488ø1) was matched to her clinical MRSA isolate. Expanded access was granted by the FDA (IND # 27264), and approval by the University of Maryland Baltimore Institutional Review Board (HP-00094882EA) was obtained. 

The patient then underwent sequential single-stage exchange of her hip spacer and a knee arthrotomy with removal of the Hickman catheters. An intraoperative dose of bacteriophage (1.2 × 10^9^ plaque-forming units (PFU)/mL) was diluted in 10 mL of normal saline with resulting titers administered into each joint of 1.2 × 10^8^ PFU/mL. While the entire 10 mL dose of bacteriophage therapy was injected into the knee joint prior to closure of the arthrotomy, the 10 mL dose for the hip was divided into 5 mL injected into the femoral canal prior to implanting the new spacer, and the remaining 5 mL injected into the hip joint prior to closure. 

Postoperative day 1, she was started on daily intravenous bacteriophage therapy (1.2 × 10^9^ PFU/mL) diluted in 50 mL of normal saline and infused over 30 min with resulting titers of 2.4 × 10^7^ PFU/mL administered. A slight transaminitis to twice the upper limit of normal was observed on postoperative day 1, but the transaminitis did not worsen with subsequent intravenous daily dosing of bacteriophage therapy, which continued for 3 days. The transaminitis returned to normal three days after stopping bacteriophage therapy. Daptomycin was continued for three more weeks, followed by Bactrim DS for three weeks, and then antibiotics were stopped. 

Two months thereafter, she underwent a permanent total hip arthroplasty with adjuvant intra-articular bacteriophage therapy (1.2 × 10^9^ PFU/mL), diluted in 10 mL of normal saline (Figure 1C). Five milliliters (2.4 × 10^8^ PFU/mL) were injected into the femoral canal prior to implantation of the permanent femoral component, and the remaining 5 mL (2.4 × 10^8^ PFU/mL) were injected into the joint prior to closure. All intraoperative bacteriological cultures were negative. Three months later, while still off antibiotics, she underwent permanent total knee arthroplasty with intraoperative bacteriophage therapy (1.2 × 10^9^ PFU/mL) diluted in 10 mL of normal saline (Figure 1D). The bacteriophage dose was divided with 3 mL (4 × 10^8^ PFU/mL), injected into the femoral canal, and 3 mL (4 × 10^8^ PFU/mL) injected into the tibial canal prior to implantation of the components. The remaining 4 mL (3 × 10^8^ PFU/mL) was injected into the joint prior to closure. All intraoperative bacteriological cultures were negative. Eleven months since receiving the first doses of personalized bacteriophage therapy, there has been no evidence of recurrence, and the patient is ambulating without a cane, able to climb stairs and driving using her operative right leg. 

## 3. Discussion

**Figure 2:**
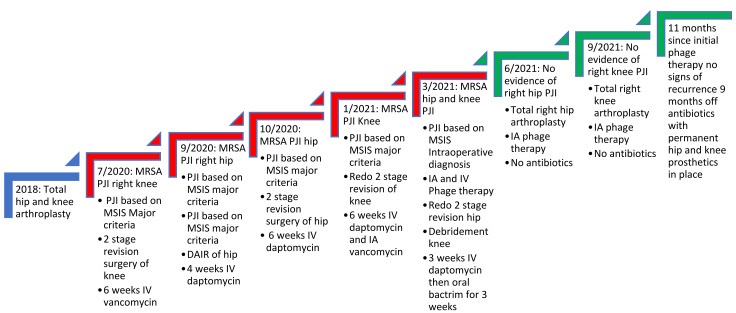
Timeline of the patient’s recalcitrant MRSA PJI of knee and hip. Red steps indicate continued infection, while green steps indicate resolution of infection as seen with no clinical symptoms and sterile deep tissue bacteriological cultures. The figure shows it was not until bacteriophage therapy was used that cure of infection occurred. IA refers to intraarticular administration. DAIR refers to debridement and implant retention surgery. MSIS refers to musculoskeletal infection society. The MSIS criteria for PJI diagnosis are well documented elsewhere [2].

When two-stage revision surgery fails to cure PJIs, evaluating the reason for such a failure is paramount to eradicating the infection. Common risk factors for failure include obesity, immunosuppression, poor wound healing and uncontrolled infections at other sites, such as endovascular infections [7]. Furthermore, poorly treated PJIs with inadequate source control can lead to uncontrolled planktonic infections presenting with recurrence of symptoms soon after the removal of the prosthesis. In this case, the patient had no risk factors for failure, nor did she have a distal source of infection, as demonstrated by no endocarditis on transesophageal echocardiogram or any niduses of MRSA infections on CT scans of the chest, abdomen and pelvis. In addition, her initial improvement on antibiotics supported adequate source control and eradication of her planktonic MRSA infection. However, within weeks of stopping antibiotics, her PJIs would recur with the same MRSA, indicating that a deep-seated recalcitrant infection was present that conventional antibiotics could not eradicate (Figure 2). 

While planktonic bacteria are responsible for the overt symptoms of PJIs, numerous other factors cause PJIs to be arduous to treat. These include biofilms, small colony variants, persister cells, formation of plasma protein aggregates and formation of small abscesses in canaliculi of bones [6,8,9,10,11]. Unfortunately, conventional antibiotics have limited ability to completely eradicate these stationary states given systemic administration of antibiotics never achieve high enough concentrations. This is supported by in vitro experiments where concentrations of some antibiotics needed to kill biofilm bacteria can be up to 1000 times the concentrations of those needed to kill planktonic bacteria [12]. Even high local concentrations of antibiotics administered with repeated intra-articular dosing do not address all the potential causes discussed above, and consequently, recurrence can occur. As seen here, the patient was only able to achieve long-lasting eradication after the use of a short course of personalized bacteriophage therapy. 

We utilized personalized bacteriophage therapy with surgery to (1) directly apply bacteriophages to the recalcitrant infection locations, (2) immediately engage bacteriophages in biofilms that had been manually debrided intraoperatively and (3) initially circumvent the unknown bacteriophage pharmacokinetics and rapid clearance that can be associated with intravenous dosing [13]. The volume of normal saline (10 mL) that was used to dilute the intraoperative bacteriophage administration is based on our experiences with intraoperative dosing of bacteriophage therapy [8]. Moreover, when prosthetic reimplantation was conducted, the volumes of bacteriophage administered were equally distributed to locations where the prosthetic was to be implanted. 

We also administered intravenous bacteriophage therapy for three days to reach locations that may be inaccessible to intraoperative bacteriophage administration, such as cortical canaliculi, distal bones and soft tissues that may have harbored niduses of infection. While no major adverse events were seen with bacteriophage administration, the patient did have a small initial increase in liver enzymes to twice the upper limit of normal that has been described elsewhere [14]. With subsequent intravenous bacteriophage administration, no further increases were observed, but this reinforces the need to follow liver function closely when using bacteriophage therapy for PJIs. 

As seen here, bacteriophage therapy is a promising adjuvant therapeutic in the treatment of chronic PJIs. Moreover, the use of bacteriophage therapy with DAIR may allow for cure without prosthesis removal, but this therapeutic also has the potential to help improve outcomes of two-stage revision surgery, especially in patients who have failed prior two-stage revision surgeries or in those who are at high risk for failure. Taking into consideration the success of two-stage revision surgery, which is approximately 85% [15], testing bacteriophage therapy with this intervention would require a large clinical trial with collaboration among many centers to adequately power such a study. However, contrary to using bacteriophage with DAIR, there would be ethical equipoise in conducting a randomized controlled clinical trial for chronic PJI comparing two-stage revision surgery to two-stage revision surgery with adjuvant bacteriophage therapy. Therefore, while the large sample size and associated financial ramifications may obstruct such a trial, it would be the lowest risk application of bacteriophage therapy in PJIs to test the efficacy of this therapeutic. 

The major limitation of this case is that the bacteriophage therapeutic was used as an adjuvant with surgical interventions, which clouds the perceived effectiveness of this therapeutic. However, the central dogma of PJI treatment is infection source control, whereby surgical interventions debulk most of the infection while also manually debriding biofilms. These interventions likely make chronic infections more receptive to bacteriophage predation [6]. In this case, clinical and microbiological cure were observed as documented, with sterile intraoperative cultures and with no recurrence of antibiotics. While the use of this bacteriophage as an adjuvant therapeutic with surgery does have promise in PJI, only well-designed clinical trials will be able to adequately determine if this therapeutic has reproducible efficacy. 

## 4. Materials and Methods

### 4.1. Bacterial Isolation

All MRSA isolates were grown on blood agar plates and then identified with the Vitek 2 (BioMerieux, Marcy-l’Étoile, France). The MRSA bacterial isolate used for bacteriophage screening was obtained from sterile operating room cultures. No other pathogens were recovered from the arthrocentesis culture or from the operating room cultures. The clinical isolate was then subcultured onto a tryptic soy agar slant that was sent to Adaptive Phage Therapeutics to identify an appropriate bacteriophage therapeutic that had lytic activity to her clinical isolate. 

### 4.2. Bacteriophage Screening, Amplification and Purification

The Host Range Quick Test was used to observe the clinical isolate sensitivities against the PhageBank *S. aureus* bacteriophages. This was conducted by growing the clinical isolate to log phase (as determined by spectrophotometric methods) and then serially diluting in a microtiter plate containing tryptic soy broth and a metabolic dye [16]. Individual bacteriophage strains were then introduced to the clinical isolate, and the interaction was observed for 48 h; bacteriophage sensitivity was measured by the inhibition of cellular respiration [16]. Bacteriophages that successfully inhibited the rate of respiration were also tested for plaquing in a double agar overlay method. The *S. aureus* bacteriophage, SaWIQ0488ø1, showed inhibition for >30 h relative to the bacterial host control and was selected for therapy, as has been used by others [17,18]. The amplification and purification are discussed elsewhere [8]. The lot of final therapeutic vials were quality control tested for titers, sterility and endotoxin levels. The results from quality control testing can be found in Table 1.

## 5. Conclusions

In conclusion, this case describes the successful use of bacteriophage therapy in a recalcitrant MRSA hip and knee PJI that had failed conventional revision surgeries. We used bacteriophage therapy as an adjuvant with surgical intervention to allow for the most successful application and consequently the best chance of cure. Bacteriophage therapy is a promising therapeutic in PJIs, but more translational research is needed to devise reproducible protocols to thereby conduct clinical trials evaluating efficacy used with either DAIR and/or with revision surgeries. 

## Figures and Tables

**Table 1:** Sterility, titers and endotoxin levels of bacteriophage used in this case.

Phage ID	Titer (PFU/mL)	Endotoxin (EU/Dose)	Sterility Testing
**SaWIQ0488ø1**	1.2 × 10^9^	<1	No Growth

## Data Availability

Data are contained within the article.
